# Dyspepsia

**Published:** 1873-09

**Authors:** 


### DYSPEPSIA.

A New York lawyer of high standing, of unusual professional ability, with a large and lucrative business, and a wife and two children, shot himself in the head the other day, at the age of fifty-four years, under the influence of great mental depression, arising from dyspepsia. Multitudes of others have destroyed life from the same cause. All diseases tend more or less to depress the spirits and to induce melancholy, without much pain, necessarily; but the sufferings accompanying dyspepsia are very great, and follow every meal, when the disease becomes confirmed. Whoever has any uncomfortable feeling after a regular, moderate meal always has dyspepsia. Different persons have different sensations, but the point to be noticed is not the special feeling; if it is uncomfortable, uniformly uncomfortable, that is dyspepsia.

Another item is, medicine has never cured dyspepsia. It is essentially a malady resulting from the inability of the system to prepare the food for imparting nutriment to the body, by making good blood; and the blood, being bad, is unnatural, and must, wherever it goes, cause an unnatural state of the part, and since this blood goes everywhere, a dyspeptic feels "bad all over;" and, as proper nourishment is not given, the dyspeptic is more or less weak, although in some cases he does not lose flesh, and the muscles of the limbs are hard—but, generally, dyspeptics are thin and the flesh is soft and flabby. The only mode of cure is in eating at regular times, of suitable food, in proper quantity.

The meals should be taken at not less than five hours apart, and in such quantity as will leave no sensation whatever sufficient to attract the attention disagreeably. It is this, that or the other kind of food which must be eaten or avoided. Dyspeptics are constantly inquiring what shall be eaten and what avoided. There is no invariable rule, for some can eat one thing and some another to apparent advantage; it is the principle which must be looked to, and that is, do not eat so as to cause uncomfortableness afterwards. We are very apt to think that it is the quality of the food, but nine times in ten it is the quantity. A particular kind of food, in small quantity, may do us good, which, if taken largely, does not only fail to nourish but causes positive suffering. It is nourishment that the system wants, and all food contains nutritive particles; but, as a very general rule, fresh lean meat, fruits and coarse breads are applicable. How much must I eat? is the universal inquiry. Not any two in a hundred require the same amount of food; hence, never weigh and measure what you eat; each must be a rule to himself, but any man may find out in less than a week what quantity of food is suitable for him by diminishing the amount gradually, until he ascertains how much he can take without any discomfort whatever. At the end of a week increase the amount by slow degrees, always stopping short of what will cause a disagreeable sensation. Efficiency will be given to the above treatment infallibly, in proportion as the patient employs himself in moderate agreeable out door activities—and, above all, such as are encouragingly remunerative. Nine tenths of all the dyspeptics are caused by irregular and too frequent eating. The foundation of almost all women dyspeptics is laid in the "teens" of girlhood, by everlasting "nibbling."

